# Book Reviews

**Published:** 1806-01

**Authors:** 


### CRITICAL ANALYSIS OF THE RECENT PUBLICATIONS ON THE DIFFERENT BRANCHES OF PHYSIC, SURGERY, AND MEDICAL PHILOSOPHY.

---

*A Medical and Experimental Enquiry into the Origin, Symptoms, and Cure of Constitutional Diseases, particularly Scrofula, Cancer, Consumption, and Gout. By* WM. LAMBE, M. D. Fellow of the Royal College of Physicians, 8vo. 5s. 6d, 1805.

THERE is something in style which fascinates the mind: Where every period runs smooth, we follow, or rather attend, the author with so much rapidity and ease, that meeting with no difficulties we reach the end of our journey almost without enquiring whether we are pursuing the right road. To illustrate our meaning, we shall offer a few uninterrupted extracts from the work before us, and subjoin some very short remarks.

The work may be divided into three parts. The first, called Preliminary Observations, contains a vast variety of matter, offered to prove that water is the source of all our chronic diseases. The second part consists of some theories concerning the four diseases mentioned in the title page, to show the probability of their arising from the deleterious properties of water, with some cases under each head, proving the advantage of the regimen recommended by the author. In the third part we have several experiments to show that it is possible arsenic may be contained in most, if not all waters in different proportions, and that the introduction of this substance into the body produces gradually those symptoms which constitute the above mentioned and other chronic diseases.

After a few general remarks, that medical writers have been hasty in imputing all chronic diseases indiscriminately to the effects of civilization, our author proceeds:

" A little attention may convince us, that it is not man only whose frame has been injured by civilization. All the animals which have approached his habitations, or have been reduced under his dominion, have also partaken of his misfortune. The domestic fowl acquires a morbid delicacy, so that the greater part of the young frequently perish. The horse, as he seems to partake much of the disposition, and to possess many of the passions of his rider, has likewise the greater part of his diseases. Like him, he is subject to inflammations, fevers, consumption, tetanus, and other maladies, very nearly resembling those of the human subject. Here mental causes, to which we are apt to attribute so much in the generation of human infirmities, are necessarily excluded. Nor can even even luxury be justly charged with these dreadful consequences, if taken in the sense in which we apply this term, to the diet and habits of mankind.

"Still we cannot but apprehend that the motions of material systems are principally dependent upon material forces, and therefore, that the principal agents in these wonderful phenomena, may be rendered the objects of our senses. If so, is it not possible to avoid them? Can the evils of social life be escaped only by renouncing its advantages and by returning to barbarism? This question is certainly the most important that can be proposed to human wisdom. I will not venture to assert, that it may be answered in the affirmative. But my senses and my understanding have utterly deceived me, if a very great improvement may not be made in the condition of man, and particularly in the treatment of some of the diseases, which have been hitherto the most intractable, by a greater attention to the composition of his diet, and especially by avoiding the application of deleterious and poisonous matter, daily introduced into the system, perhaps in many ways, but principally, and most abundantly, under the attractive and unsuspected form of Water.

"Waters are divided, by chemical writers, into two great classes, the *oeconomical* and the *medicinal*. The former (it is with these only that we have any concern in this place) being such as are commonly applied to domestic purposes, have been supposed to contain nothing more than very minute quantities of well known salts. As these salts, when taken internally in moderate quantities, produce no bad effects on the body, they are deemed, and it would seem very properly, to be nearly inert and wholly inoffensive in the very diluted condition in which they are thus received. Such being the doctrine of chemists, the most eminent in their art, it is not at all surprising, that physicians have, in general, entertained little or no apprehensions from the indiscriminate use of the *oeconomical* waters; and that, whatever may be the suspicions of a few of the most judicious, their apprehensions have been too vague and too little supported by experiment, to have had any influence whatever upon the practice of the profession or the habits of the public.

"In the numerous experiments which I made on a great variety of common waters, with the view of determining whether, having been in contact with lead, they contained any of the metal in solution, I could not but perceive this general account of their contents to be very imperfect, and felt no small degree of astonishment at the negligence with which the subject had been treated. But though convinced that many waters possess metallic impregnations, which elude detection by the ordinary methods of examination, I felt only a vague apprehension, that this might render them not entirely salubrious: still less had I the smallest suspicion that any matter might be extracted from them of a deleterious nature. The following circumstance incited me to attempt a more full and laborious investigation of the properties of common water, which has has convinced me, that it is to be reckoned amongst the substances which have the most direct and powerful influence on the animal economy, both in health and in disease.

" A lady was occasionally afflicted with very severe pains of the stomach, when she lived at a particular house, which had repeatedly left her upon changing her residence. Unable to account for this circumstance, she requested me to examine the water used by the family. It was well tasted, but it had been observed to make the teeth dark. I used the methods I have described in another place for the detection of metallic matter, but to no purpose. Not being able to divest myself of the suspicion, that some noxious substance must be contained in this water, I evaporated a small portion of it to dryness and tasted the residuum. Now I observed that, though it hardly impressed the tongue with any other taste than the bitterness of the deliquescent salts, there was a peculiarly disagreeable sense of constriction excited in the fauces, which remained there fixed for a long time. The impression was clearly metallic. Though my mind revolted at the suspicion, I thought I perceived a strong resemblance between this impression and that excited by arsenical salts. I washed out the deliquescent matter, and put the remainder, mixed with a little charcoal powder, between plates of copper, which I exposed to a red heat. The copper received a white stain by this process. A little arsenic was exposed to the same treatment between similar plates. No difference could be observed between these stains in each experiment, unless that the impression made by the residuum of the water, was the more distinct of the two. Thus was a great degree of probability added to the suspicions I had previously entertained.

" Amazed at a result, so strange and unexpected, a croud of reflections could not but rush upon my mind. What! is it possible that human beings can be daily swallowing the most virulent of poisons, without suspicion and almost without complaint? Those who have resided at this place have not been singularly unhealthy, and some have arrived at the ordinary period of old age. The fact then cannot be solitary. Is not this the very dæmon, which, for so many ages, has tortured mankind; and which, usurping the sensorium, has corrupted, under a thousand forms, both the mind and body? the evil spirit, which has augmented the wants of man, while it has diminished his enjoyments? which has exasperated the passions, inflamed the appetites, benumbed the senses, and enfeebled the understanding? which has converted his fine form into a storehouse of diseases, has blasted the flower of his offspring, and has brought even the strongest of his name to an untimely grave."

The following is extracted from the second part of the work:

" Under this form, we call the disease *Scrophula*; a form universally acknowledged as a common precursor and parent of pulmonary consumption. As puberty advances, the activity of the absorbent vessels diminishes with the utility which gave birth to it. We are now presented with an appearance demonstrating almost to the the eye, that this disease is the consequence of excessive stimulation. Young people are observed to grow with extraordinary rapidity, and upon reaching their full stature, the pulmonary symptoms appear. In these cases, every body remarks that such subjects have overgrown their strength.

" At puberty this morbid activity becomes concentrated, as it were, to a point. All the organs have attained perfection, and all the actions are directed to the purposes of conservation. The morbid activity becomes confined to the excretory organs, of which the lungs is the principal. Through these is the stimulating matter perpetually eliminated; but being as constantly renewed, the viscus becomes injured by excessive action, and the vital powers sink under the perpetual irritation and constant exhaustion.

" If after a certain age, the system becomes less subject to consumption, it is because the excreting organs are less active, and the mobility of the system is greatly impaired. Under these circumstances, large parts of the system are apt to become torpid or paralytic. Therefore if, after the consumptive period, the vital powers begin to give way, the forms of disease incline to dropsical swellings, congestions in the bowels, *leucorrhœa*, hæmorrhoids, asthma, palsy, &c. The colour and countenance alter, inclining more and more to that which is termed melancholic. Thus, the consumptive systems must in some respects be deemed the most perfect, since they perish from the excess of their own actions. In the chronic diseases of more advanced periods, the powers themselves become impaired, and are in the end destroyed."

Some cases are added of various success, but sufficient, in our Author's opinion, to sanction his theory of the causes of these diseases.

Even Cancer, Dr. Lambe cannot help considering as deriving its origin from the same source as other constitutional diseases. Among other arguments the following passage contains a group of them condensed:

" It has fallen in my own way often to meet with cancer and consumption in different members of the same family. Cancer and gout are united, not uncommonly, in the same subject.

" Mr. Howard has insisted too on the affinity between cancer and leprosy. That between cancer and elephantiasis has been observed many ages ago by Galen. On the latter disease he has made a remark, which may be transferred to the former with strict justice, supposing this affinity to be real. He presumes the elephantiasis to be the joint effect of climate and diet. Hence, he observes, that it is common in Alexandria, but in Germany and Mysia it is rarely met with. Among the *milk-drinking* Scythians (γαλακτοπότας Σκυθαις) it is almost unknown. These Schytians lived in about the same latitudes as the Germans and Mysians, and therefore, the efficacy of their diet is the more striking."

Some cases are annexed to show the efficacy of distilled water in this disease, and also in gout, in which our author finds sufficient authority authority to support him in the theory that this disease arises from morbific matter. Of the nature of the matter his opinion is of course different from former writers.

"Whilst, therefore, I contend for the existence of a morbific matter in gout, I see no reason for supposing it to be at all peculiar to gouty subjects, or any other than the same septic poison, which I believe to lay the foundation of other constitutional diseases. And the relief received from the paroxysms I presume to be due, not to the inflammation of the feet, but to the changes induced by the process of fever."

These opinions, as in former instances, are illustrated with cases: and this part of the work concludes with some lively reflections, which it is impossible not to peruse without admiring the happy talent which our author possesses in writing.

The experimental part of the work consists of: 1st. An analysis of arsenicated manganese; 2d. Of the properties of the ashes of animal matter; and, 3dly, Of water. Our author does not pretend to have discovered arsenic in either animal matter or water. But he found so many of the same properties, or rather of the same phenomena, resulting from his experiments on them, and on arsenicated manganese, as to satisfy him of the existence of arsenic in those other substances.

Having given as candid an account as we can of this work; we are aware that most of our medical readers may be contented with those suggestions which have arisen in their own minds during the perusal of our extracts. But we wish them to be considered, as they really are, very imperfect, without a full view of the other numerous arguments and illustrations produced in the work. Having therefore perused the whole, we shall take the liberty to offer our opinion, which we do with some reluctance, because we cannot doubt the good intentions or industry of the author.

And, first, we cannot clearly understand if these diseases arise from the impurity of common water, why man in his more civilised state, or other animals as they become domesticated, should be more afflicted with them. Man, in his residence in this great town, is certainly not a water-drinker; and our author admits that the precipitation by fermentation somewhat improves, though it does not entirely destroy the bad properties of that fluid. As to the other animals, they fare for the most part better when under our care than in a state of nature, as we are careful to procure many of them the water which is found most agreeable to their divestive organs.

We give our author every credit for the sympathy with which he speaks of our common nature: "If, says he, the origin of the sufferings of mankind cannot be discovered in the operation of the matters which are applied to the human frame; it is to be feared that the condition of the race must be considered as utterly hopeless." Melancholy as this consideration may be, we are at a loss to know what remedy our author would propose; for if it is proved ed, that not only water, but animal and consequently vegetable substances, contain essentially arsenical matter (page 257), and if this matter is the sceptic principle, the foundation of all these chronic diseases, as long as we eat and drink, we must submit to eat and drink arsenic. Nor can we easily understand how we are to escape by butter-milk and potatoes, which are recommended as the source of health and beauty, on the grave authority of Doctor Adam Smith. But we hope our condition is not quite so bad; even admitting that arsenic may be found so universally diffused, and admitting, what no one will deny, that arsenic is poison. It is asked, (p. 154) can that which is a poison in a state of concentration, be made innocent by dilution? For our own parts, we should answer, Yes. Nor do we recollect such a position being doubted till now.

We shall conclude with a few general remarks for our author's future consideration, should this work arrive at a second edition. We conceive too little regard has been paid to climate and too much to water. Though men live pretty much the same in most climates, yet scrofula is only known in the colder and more uncertain regions. Whatever analogy there may be between cancer and leprosy, it is certain that one of them is a disease of cold and the other of warm climates. The observation of Galen, which we have quoted above, refers much more to climate than diet. The climate of Egypt has always been famous for elephantiasis, though we have no reason to suppose that the diet has been materially different in Mysia and Germany, where the disease is rarely met with. The milk-drinking Scythians inhabited a still colder climate, and probably owe their exemption to that, more than to their diet. We are told indeed, that "these Scythians lived in *about* the same climate as the Germans and Mysians, the efficacy of their diet is therefore the more striking." We conceive that our author's qualifying *about*, is pretty equivalent to the difference of *rarely met with* in one instance, and *almost unknown* in the other.

After all, we must admit that the work before us shows extensive reading, great memory, much candour to those from whom the author differs, and is adorned with a style that would not disgrace the author of the sublime and beautiful.

---

*Commentaries on the Treatment of Scirrhi and Cancers, from the earliest Period to the present, for the purpose of pointing out and establishing a specific for those Diseases on rational and scientific Principles. By* WILLIAM THOMAS, *Member of the Royal College of Surgeons,* 8vo. pp. 47.

"NOVELTY, says our author, seems to be the great excitement in the present medical pursuits, and the hope of discovering new resources, more desirable than in those already known."

Feeble as these hopes have hitherto proved, we cannot encourage any thing that would lessen so laudable a pursuit. Mr. Thomas is of a different opinion, and though he admits that "no one will will dare to detract from merit so happily engaged; yet the object, he adds, may probably be better promoted by gleanings from the old reapers, than by immediately treading the footsteps of the new." Alas, if it could be attained in either way, who would not rejoice at such a prospect? or who would differ about the road that might lead to such a discovery? But, to pursue the allusion. Are we to glean after those who acknowledge the produce of their harvest always proved unprofitable? or to follow the footsteps of such as confess themselves ever in the wrong?

"Such, however, continues our author, has been my opinion, and such has been my practice in forming the following pages; and *whatever novelty may be found in them*, has sprung from the deep and obsolete sources of antiquity fostered by reflection and confirmed by practice.

"Opinions strongly imbibed (as those of the author are supposed to be) are said to produce too sanguine and flattering conclusions. I hope my mind is protected from such flattering delusion, for I confidently believe the practice I have endeavoured to elucidate, will give to practitioners a resource in these deplorable cases, *which they have for too loug a period been deprived of;* and that the unfortunate sufferers who daily shrink from the horrors of the knife, will chearfully yield to this practice, which, when under judicious and scientific management, will be directed to the happiest purposes."

What will our readers say, when they find they are to be deprived of these resources a little longer. Unkind Mr. Thomas! but, perhaps, there is a doubt, whether this "judicious and scientific management," so necessary for the success of this recondite practice, is sufficiently to be depended upon in the generality of us his readers. Should our author entertain such an opinion of his weaker brethren, it is further to be regretted, that all his grand discoveries are still withheld. This first part contains indeed some Latin quotations from Vogelius, Aëtius, and a few others, and also some remarks from the moderns. By these it appears, that all the unsuccessful attempts of the former have been renewed by the latter, and, as might have been expected, with similar consequences. There is, indeed, one little note, which contains an aphorism we do not remember to have seen delivered any where else with the same solemnity!

"Sleep, says our Author, seems to have been considered peculiarly necessary. Somnus perutilis est; itaque nisi naturæ sponte obrepat arte conciliandus." Is this among the "novelties sprung from the deep and obsolete sources of antiquity, fostered by reflection and confirmed by practice?" To be short, if Mr. Thomas has any thing to communicate, we sincerely encourage him in informing the world. If he only follows the example he so much commends in Justamond, he will do a service to science by showing the full power of popular remedies, or how little is to be expected from them. We therefore withhold all further remarks till we see his Second Part.

---

*Inoculation*

*Inoculation for the Small-pox vindicated, and its superior Efficacy and Safety to the Practice of Vaccination clearly proved.* By George Lipscomb, Surgeon. 8vo. pp. 44. London, 1805.

We wish we could give some further account of the author of this *eloquent* little pamphlet; but he has not thought it necessary to favour us with any description of himself, excepting the general title of Surgeon. Whether of the London College, or attached to any charitable institution, we are entirely left in the dark. Three things however appear, before we enter on the performance. First, that the author found no other writer from whom he could select a motto so apt for his purpose as from himself; yet, on perusing the passage, it seems neither perfectly new nor indeed so recondite as to have been overlooked by others. However, it contains a certain truism, whatever may be the case with the work to which it is here prefixed. It assures us that error, printed in Roman characters, continues to be the same when printed in Italics,—not only the same, but unchanged in its nature and effects. We shall transcribe this pithy little motto.

"Error has been frequently sanctioned by the reputation of great names; but it still continues to be *error*, unchanged in its nature and effects by the most general reception which the world may have been induced to give it, in consequence of a too ready submission to the dogmas of authority."—*Lipscomb on Asthma.*

The next thing that met our eye was the author's gallantry in dedicating his work to a Lady. It is true, the Lady is dead; but this shows our author's disinterestedness.

The succeeding page informs us that Mr. Lipscomb is not only a great writer but also a great traveller. Besides the Treatise on Asthma and another medical work, he announces in due form his Tours to the South West and North of England, and South Wales!

The work itself begins with some remarks, not indeed entirely original, on the liberality of the age in attending to whatever is new in the improvement of Physic. After this, we are told of the perfect satisfaction Dr. Jenner and others expressed, when they found that such as had been vaccinated, could not receive the small-pox by inoculation, and also the ingenious suggestions of Mr. Laurence, "of the seeds of variolous infection being only smothered, or their activity suspended and not eradicated, by the *saturation*, as he termed it, of the juices with vaccine virus, and that after a certain time, and the cessation of such cause of suspension, some other cause might arise capable of exciting them into activity." If this long sentence about the *smothering of seeds;* about *suspending the activity of seeds;* about *eradicating seeds; the activity of seeds,* by *the saturation of the juices with vaccine virus, &c.*; if it means any thing, we suppose the intention is to show, that cow-pox might be only a temporary security against the variolous infection. If this is really the author's meaning, we wish, in the next edition, he he would give it us without that figurative language which he has invented or adopted.

After this our author states the question, as he conceives, with fairness, and in a manner he wishes every practitioner had confined himself to. We cannot however join in this wish, as we think there is more in the question than he proposes; for it is inconceivable how carefully this gentleman and all the other anti-vaccinists overlook the question concerning the diffusing of variolous infection by the practice of inoculation. But we shall give Mr. L's own words, for a reason which our readers will perceive at the conclusion of our quotation.

"Thus it appeared (says our author) that the grand question before the public, and now that universal attention had been excited to it, which was fairly at issue, was, Whether vaccination was an equally safe and certain, and in any degree a milder, preventive of the small-pox than variolous inoculation properly conducted?

"If practitioners had confined themselves to this simple question, much valuable time and great and laborious exertions might have been saved; but many, who dedicated themselves to this investigation, seem to have been more attentive to the *peculiar properties* of the new disease than to its *comparative merits;* many, in their enthusiastic approbation of the discovery, seem to have almost forgotten the practice to which vaccination was to be opposed, and employed themselves in panegyrising the benevolence and philanthropy of the discoverer, rather than in fairly appreciating the value of his suggestions. Many among those, who were eager to introduce vaccination, spoke of it in terms which referred to the ravages of the small-pox naturally; and overlooked the beneficial influence of inoculation, which, when judiciously performed, had been found *indeed* capable of disarming that terrific disease of its primeval horrors.

"It is but simple justice to Dr. Jenner to remark, that the Doctor stated, in the most unequivocal manner, that he thought "much precaution necessary in the progress of the enquiry." Mr. John Hunter excellently observed, on a different occasion, that no man was fit to make *any* experiment who had not made *many;* by which I understand him to have thought that those accounts of experiments which are given to us by persons not frequently accustomed to minute investigation and analysis, are seldom to be depended on. Whatever Dr. Jenner or Mr. Hunter *might think* or *may have said*, the experiments in vaccination have been, in many instances, conducted by strange agents. Country clergymen, farmers, and old women, have been made the instruments for ascertaining the consequences of this important revolution in medical science. I would not be misunderstood as intending to give offence to either of these classes when I say, that however respectable, useful, and necessary, they may be in their several stations, it is impossible that any of them should have been properly employed on on this occasion; and greatly as I venerate and admire the learning and the moral worth of the clergy, greatly as I esteem and regard the honest and beneficial industry of the farmer, I cannot help thinking that less mischief has been done by the third description of persons above alluded to, in the practice of vaccination, than by either of the other—because *they* have *never published on the subject.*"

How much easier it is to give advice than to take it!

---

*Observations on the Nature and Cure of Fevers and Diseases of the West and East Indies, and of America, with an Account of Dissections performed in these Climates, and general Remarks on Diseases of the Army.* By THOMAS CLARK, Surgeon. Octavo. pp. 257. Edinburgh.

THIS work, though certainly entitled to our notice, has hitherto escaped it, probably from not being published in London, and from some omission in the manner of introducing it.

In the Preface the author informs us, that his intention in appearing before the public is not so much to extend the limits of science, or diffuse the knowledge of any thing extremely new in the theory or practice of medicine, as to vindicate his own character against an attack which has proved highly injurious to his health and fortune.

This subject certainly deserves attention, not only as it relates to Mr. Clark, but as it respects the arrangement of a branch of the army service, scarcely less important than tactics, and perhaps oftentimes even more so in the warmer climates.

"In 1798, when the author was at Colombo, in the island of Ceylon, in the East Indies, attending his duty as surgeon of the 19th Regiment of Foot, he received orders to write *Case-Books,* or a systematic detail of the whole symptoms, &c. of all the patients that should come under his care, and of the medicines administered to them. These orders were issued by the Physician and Inspector General of Hospitals at Ceylon, and Physician to his Majesty's Forces in India, and were addressed to all the surgeons in the service of Government in India, and of the East India Company in Ceylon.

"In these enervating climates, the European temperament soon becomes too relaxed to admit of unnecessary exertions. It uniformly happens also, that the medical officers appointed by Government, are barely adequate, in point of numbers, to fulfil the ordinary duties incumbent on them. Hence it happened, that not one of the Company's surgeons at Ceylon, and none of the King's surgeons in all India, obeyed the above orders, excepting only the author, for a time, and Mr. Christie of the 80th Regiment.

"At the date of the orders, the author's own health was in a very imperfect state. He arranged the duties of his office, however, in such a way as might enable his assistants to write the

Case-Books required. They accordingly did so for one month; at the end of which period the books were transmitted to the Physician General.

"In a few days thereafter, that gentleman thought fit to transmit to the author a long and elaborate dissertation upon his Case-Books, in which he was pleased totally to reprobate the medical practice reported in them. He went even so far as to assert, that one of the patients must actually have been poisoned by the medicines he received; and he required, that the mode of practice should be entirely altered. Had not this dissertation unfortunately been lost, in consequence of the author's incapacity to attend to his affairs at the time he left India, it would have been annexed to this work.

"The author's assistants had all along considered the obligation to write the Case-books as an intolerable piece of drudgery, when added to their other duties, which they accounted abundantly severe; and they now resolved to cease to obey an order, which was disregarded with impunity by almost every surgeon in India. The author did not disapprove of their resolution, as he was satisfied that they had enough of other more pressing employment. The consequence, however, was, that in a few days, when the Physician-General visited their hospital, and found that no Case-Books were kept, he put the author under arrest, upon the charge of disobedience of orders.

"A trial by a Court Martial was of course expected to be brought on in seven days at farthest. When that period had nearly elapsed, and the author still continued under arrest, without any steps being taken to bring him to trial, he himself made application for that purpose to the Governor of the Island, the Honourable Frederic North; and in the mean time, he wrote, and transmitted to the Medical Board at Madras, an account of his mode of practice in his hospital, and likewise of his theoretical opinions.

"Still, however, he remained under arrest, and most unaccountably no trial was granted, though repeatedly requested and solicited, in every form, during more than three months. At the end of this period, his arrest was removed, but without any trial.

"The effect of being kept in this unhappy state of suspense, unfortunately was, that his health became greatly impaired, and by the time his arrest was removed, his friends found it necessary to provide for him a passage in one of the cinnamon ships, (The London, Capt. Lukin,) and to send him to England, to afford him some chance of recovery.

"Even on board this vessel, the same influence, which had already injured him so deeply, appears still to have pursued him, and soon rendered his situation so unpleasant, that when he arrived at St. Helena, he took a passage in an American ship, bound for Boston, being unable to procure one in any of the other homeward-bound Indiamen.

"In a short time after his arrival in this country, the author was was under the necessity of either returning to India, which his health did not permit, and for which he had little inclination after the treatment he had there experienced, or of retiring upon what is called half-pay, as he was not permitted to dispose of his commission. The result of the whole has been, that, after having served His Majesty in all the quarters of the globe, at the great hazard of his life, and with much loss of health, he has been compelled to exchange a very lucrative appointment for the miserable allowance of two shillings a day."

We have thought it right to copy this statement as we find it. Whatever can throw light on the arrangement of the medical department should be brought before the public, and though, like all human institutions, some imperfections must ever remain, yet it is desirable that the service should be so far attended to, respecting the rank and situation of the faculty, as to induce the best, and the best informed men, to engage their whole time and thoughts in the duties of their profession.

The first chapter of the work contains remarks on the nature and cure of fever. In this are some very good practical hints, and to them we wish our author had confined himself. His theory is to us quite unsatisfactory; for which reason we shall pass it over, because it would not be fair to attempt refuting it without transcribing the whole.

The second chapter on diseases in warm climates is less exceptionable in these respects; that is, the theory is more incidental, and occupies less room, and the practical remarks are much more extensive.

In the third part the author begins to reduce his general remarks to particular diseases. Inflammation of the lungs occupies the first chapter; the symptoms of which, in warm climates, are well described: as to the remedy, hitherto, we believe even the various fashions of medicine has not produced any change of opinion.

The next chapter is on Dysentery. This also is replete with very useful practical observations. One of these we must not omit, as it appears not only entirely our author's, but replete with sound judgment, a proof of great attention to his duties, and, as he assures us, highly successful.

"Having, in violent cases, often found the remedies now described, or any others that I had tried, ineffectual, I at last had recourse to the use of emetic substances in the way of injection. I did not adopt these, however, till I had reflected very seriously, and reasoned very fully on the subject. The other remedies, already mentioned, except injections, were administered at the same time.

"From much experience, I do not hesitate to assert, that they have been, and I believe I may venture to say will be, found extremely beneficial in dysentery.

"It appears to me more than probable, that they will also prove useful in cases of piles, or, in short, in all kinds of inflammation affecting the rectum and parts adjoining.

"When given early in the disease, they generally afford immediate relief, and sometimes one or two injections effect a cure. When they have not been used until the advanced stages, the patients experience more uneasiness from them, particularly on their being first thrown up; but if they can be prevailed upon to keep them for a minute or two, the uneasiness in a great measure ceases, and they often are able to retain them for a considerable length of time.

"The manner in which these injections operate, is for the most part as follows.

"In the incipient stages of the disease, even when attended with violent pain and tenesmus, and all the more violent symptoms of this complaint, immediate relief is almost constantly experienced from them; and they are commonly retained for a considerable length of time with little or no uneasiness. At length, an effort to go to stool comes on, and several copious natural evacuations, mixed with mucus, are procured; and in the more violent cases, several evacuations of slime, or mucus alone, or intermixed with blood, succeed to the natural stools, accompanied with little or no straining. After this, the patient commonly remains for a number of hours, without any symptoms of disease; and in some instances it does not return.

"Those injections do not appear to occasion vomiting, or even to increase the irritability of stomach, that may have previously existed. They probably assist in increasing perspiration. But from being in the constant practice of keeping up a copious perspiration, I cannot so well judge of this part of their operation. However, I do not believe that they operate very powerfully in that way; at least, in some cases I have found it impossible to produce a copious perspiration by ipecacuanha, both in the form of injection, and also at the same time given by the mouth, in considerable quantities. In such cases, I have very seldom found James's powder and laudanum, given in small and repeated doses, fail in producing the desired effect.

"The salutary effects of these injections appear to me to depend chiefly upon their exciting a copious secretion of mucus from the internal coat of the great guts, and thereby removing the inflammation affecting them.

"I have known a few ounces of this injection give immediate and permanent relief in several instances of very painful inflammatory affections, about the extremity of the rectum; a copious secretion of mucus, resembling the whites of eggs, being produced.

"I generally have given two, and sometimes three, in twenty-four hours. The best general rule, I believe, is to administer injections, whenever the more violent symptoms of dysentery return, or threaten to do so.

"Strangury, which frequently accompanies violent cases of dysentery, sentery, will be found very seldom troublesome, when these injections are used; the reason why it is not so, must appear obvious to every one.

"The form of injection which I have found to answer best, has been about three drachms of ipecacuanha root, bruised, and boiled in a quart of water down to a pint, and given at once as a glyster.

"From ten to twenty grains of tartar-emetic, dissolved in a pint of warm water, will produce nearly similar effects.

"With regard to prognostics in this complaint, let it suffice to say, that it is always a dangerous disease, when accompanied with much fever. The more violent, and the more frequent, and more bloody, the evacuations are, the greater will be the danger. When cold sweat, hiccup, and a frequent feeble pulse take place, along with cessation of pain, and copious bloody stools, though perhaps less frequent than before, death may soon be expected; gangrene or mortification then undoubtedly having taken place.

"After the disease has continued a considerable time, and perhaps assumed more the appearance of diarrhœa than dysentery, and if, at the same time, the patient should be much reduced, things are to be looked upon as very unfavourable."

Part the fifth contains remarks on the liver complaint. It is very surprising, considering how frequently this complaint occurs in a country abounding with medical practitioners, who have more advantage in learning the diseases of the climate than any others of the world, that we should have so few accounts, and those accounts so unsatisfactory. The present contains many little facts we do not remember to have seen before, leading to practical inferences highly important; the theoretic reasoning with which it is interlarded might have been spared, for though it seems further to explain all the phænomena of the disease, we think this might have been done as well by a bare recital of facts. But though we approve every part of our author's descriptions, we cannot help fearing lest, as in most other cases, his theory, if it is erroneous, may lead to an erroneous practice. "May not, (we are asked) the salutary effects of mercury in liver complaint depend considerably on its producing a determination to the surface, and thereby diminishing the superabundant additional quantity of blood, and consequently enabling the biliary organs to perform their proper functions?"

It may be thought by some, that if we agree in the practical object of giving mercury, it is of very little consequence what our reasons may be for it. But this is far from the case. When Sydenham introduced his invaluable practice of exposing his patients to cold air during the eruptive stage of small-pox, he at the same time proposed a theory so directly contrary to his practice, that his warmest admirers in adopting the first lost sight of the last. Thus, if we should admit with our author that the whole advantage from the use of mercury is derived from the perspiration excited by it, we shall be apt to trust to other diaphoretic remedies, which, as far as our present knowledge extends, have always proved unequal to the cure of this formidable disease.

In the next division of the work, our author appears as an army surgeon's mate in North America and the West Indies. We have a number of miscellaneous remarks on the diseases of both those climates. As Mr. Clark, during the greater part of this time, was acting partly under the directions of others, we have an account of the opinions of many well-known authors. This shows us the modesty of the writer, and his industry in acquiring knowledge from every source. But there seems to us a timidity in his practice, which may well be excused from the rank he then held. We conceive also, that his perpetual reference to diaphoretic remedies, argues a want of research which at present he would correct. When fevers, or acute diseases of any kind give way, perspiration usually follows. But that perspiration is the consequence, and not the cause, of the termination of the disease; accordingly, we frequently find it described as induced with more facility by the same remedies after bleeding than before it.

After this we find our author acting as regimental surgeon in Ireland, and meet with many useful practical remarks on the diseases of soldiers in Europe. This part closes with further illustrations of the theory of fever. In every part of our author's narrative we see, in a most lively manner, the disadvantages described by Dr. Jackson, as attending general hospitals, in comparison with attaching the sick to their own regiments.

The next part of the work is almost entirely practical, and abounds with useful cases and dissections. The scene of action is principally in the East Indies, and the voyage thither. We shall not transcribe any part of this section, because we wish such practitioners as are interested in the subject to peruse the whole.

At the end of the volume are a few observations on the army medical department, which, as they are short, pointed, and on a subject particularly important in the present state of the country, we shall transcribe without adding any comment.

" Having had occasion, in various parts of the preceding sheets, to make rather unpleasant remarks concerning the Military Medical Department, I shall shortly state what I consider to be the present condition of that truly important body of men, and then leave my readers to judge of the propriety or impropriety of my reflections thereon.

" This department consists of the following classes or orders of medical men.

" 1st. Medical Mates.—These are gentlemen who are supposed to be acquainted with the compounding of medicines, and, at the same time, are qualified to act as clerks to the others. However, I believe it now and then happens, that such gentlemen are occasionally intrusted with the sole care of sick. The impropriety, nay, even ———, of intrusting valuable lives to such unqualified characters, I think, must appear in a glaring light to every one.

" When

"When in the West Indies, I well recollect, that a young man belonging to Barbadoes, who was for some time employed as an assistant-surgeon in the general hospital there, wished to be appointed a regimental mate. An examination was deemed expedient; but, as the gentleman was completely ignorant of Anatomy, he was rejected. Before that period he had the charge of a very considerable number of brave soldiers.

"The garrison mate of Barbadoes, likewise, never had been out of the West Indies; so his opportunities of acquiring medical knowledge could not have been very great.

"2d. Assistant Surgeons.—These are gentlemen, who have undergone an examination on surgery and pharmacy. From this class, regimental surgeons, staff surgeons, apothecaries, purveyors, and inspectors of hospitals, are generally derived. In short, with the exception of physicians, (and some of them are occasionally taken from this class) they may be considered as the effective body of the medical department.

"Every one knows that the chief duty of regimental, as well as assistant surgeons, is that of physicians; and unless in time of war, they seldom have occasion to perform capital operations. Is it not, therefore, highly improper to appoint those gentlemen to act as physicians, without having any proofs whatever of their being qualified for such a truly important office.

"The *next* class are physicians.—They, I believe, when they enter the service, are generally young, and possessed of what is commonly called interest. I imagine they, for the most part, look with a single eye towards the half-pay. If I may be allowed to judge from what I have seen, the duty of those gentlemen is in general very easy, as they almost always have assistants under them, who in fact frequently do the whole of the duty.

"*Lastly*, The Medical Board.—If my information is right, these gentlemen are qualified for their situations, abstractedly speaking, as medical men. But owing to their unacquaintance with the intimate nature of the service, from not having served themselves, regulations are made by them, which, in many respects, must be inconsistent with the good of the service.—I shall, therefore, beg leave to suggest some improvements upon the present Military Medical System.

"In the *first* place, I would propose, that no medical man should be admitted into the army, without having undergone strict examinations on *every department of Medicine.*

"2dly. As men of the above description could not be procured, unless ample encouragement were given them, I would suggest the propriety of granting assistant-surgeons the rank and pay of captains; regimental surgeons that of field-officers, &c.; and that after twenty years service, they might be allowed to retire on *full pay* for life.

"3dly. That promotion should go on according to seniority; and that the Medical Board should consist of a certain number of senior surgeons.

"Lastly,

"Lastly, With all due respect to the military and medical gentlemen of His Majesty's service, I would think it advisable that a system should be adopted relative to the management of hospitals, on the same principles with that existing on the Madras establishment.

"By the regulations of the Madras Medical Board, it is made the interest of regimental surgeons to preserve the lives as well as the health of the soldiers. A daily allowance is given for each man, and officer in the regiment, sick or well, in order to provide bedding, attendants, &c.

"This plan has been productive of happy effects in India; it not only having been the means of saving many lives, but also of a very considerable sum of money annually.

"Such an innovation as now pointed out, at first sight may appear to be attended with additional expence. However, I am firmly of opinion, that the expence of paying the medical department would even be much less than it is at present, and the immense number of lives that would be preserved is incalculable. At present, young physicians, and people of a similar description, bear very heavy on the half-pay list. But if things were arranged in the manner I have suggested, this list would soon be reduced.

"I regret that the limited nature of the present undertaking does not admit of my entering at full length on this subject; so I shall stop, and resume it at some future period."

Such is the nature of this miscellaneous work, with which we have made very free, but which, we scruple not to say, abounds with more practical information than most medical books of the size; we however recommend it to be read, as indeed all medical books should be read, more to furnish hints and information, than absolutely to direct the practitioner; to assist his judgment, while he thinks for himself, however similar the cases may appear that come before him.

